# Structural basis for gating mechanism of Pannexin 1 channel

**DOI:** 10.1038/s41422-020-0313-x

**Published:** 2020-04-13

**Authors:** Luqiu Mou, Meng Ke, Mengxiao Song, Yuanyue Shan, Qingjie Xiao, Qingting Liu, Jialu Li, Ke Sun, Lei Pu, Li Guo, Jia Geng, Jianping Wu, Dong Deng

**Affiliations:** 1 Division of Obstetrics, Key Laboratory of Birth Defects and Related Disease of Women and Children of MOE, State Key Laboratory of Biotherapy, West China Second Hospital, Sichuan University, Chengdu, 610041 Sichuan China; 2 School of Life Sciences, Westlake University, Hangzhou, 310024 Zhejiang China; 3 Institute of Biology, Westlake Institute for Advanced Study, Hangzhou, 310024 Zhejiang China; 4 Key Laboratory of Structural Biology of Zhejiang Province, School of Life Sciences, Westlake University, Hangzhou, 310024 Zhejiang China; 5 Department of Laboratory Medicine, State Key Laboratory of Biotherapy, West China Hospital, Sichuan University and Collaborative Innovation Center for Biotherapy, Chengdu, 610041 Sichuan China

**Keywords:** Cryoelectron microscopy, Cell death

Dear Editor,

Pannexin 1 (PANX1) plays extensive physiological roles across diverse fields of biology, including cell death,^1,2^ inflammation,^3^ cancer progression,^4^ and neurological disorders.^5^ As suggested by biochemical analysis, PANX1 forms an oligomeric channel for the facilitated diffusion of ions and large molecules across the plasma membrane upon activation.^6^ However, the underlying molecular gating mechanism of PANX1 and the structure of human PANX1 are still elusive. In the current study, we report the cryo-EM structures of full-length and carboxyl-terminal (CT) tail-cleaved human PANX1. Combined with single-channel electrophysiological study, we identified the key residues involved in the gating of PANX1.

We purified full-length human PANX1 and solved the structure using cryo-EM (Supplementary information, Figs. S1 and S2). Among the classes of 2D particle images, the images representing the top views or bottom views exhibited seven obvious subunits in each detergent micelle. This observation differs from the previously proposed hexameric assembly of PANX1.^7^ Finally, the map was refined to 3.1 Å resolution (Supplementary information, Table S1 and Figs. S2–S4). The local resolution of the extracellular region reached ~2.8 Å, which allowed us to build residues with unambiguous assignment of side chains (Supplementary information, Figs. S2 and S4). The model we built contained an almost intact extracellular region, transmembrane domain, and partial intracellular region. Three fractions (N-terminal, 1–12; 158–193, between TM2 and TM3; and C-terminal, 355–426) were missing in the final model (Supplementary information, Fig. S5). The overall structure of human PANX1 presents a novel heptameric assembly (Fig. 1a), which is distinct from gap junction channels (connexins and innexins)^8,9^ and functionally related channels, such as CALHM and LRRC8. Interestingly, the protomer of PANX1 shares a similar fold of four transmembrane helices with the aforementioned channels (Supplementary information, Fig. S6). The distinctive structure of PANX1 implies its different substrate recognition and gating mechanisms.

**Fig. 1:**
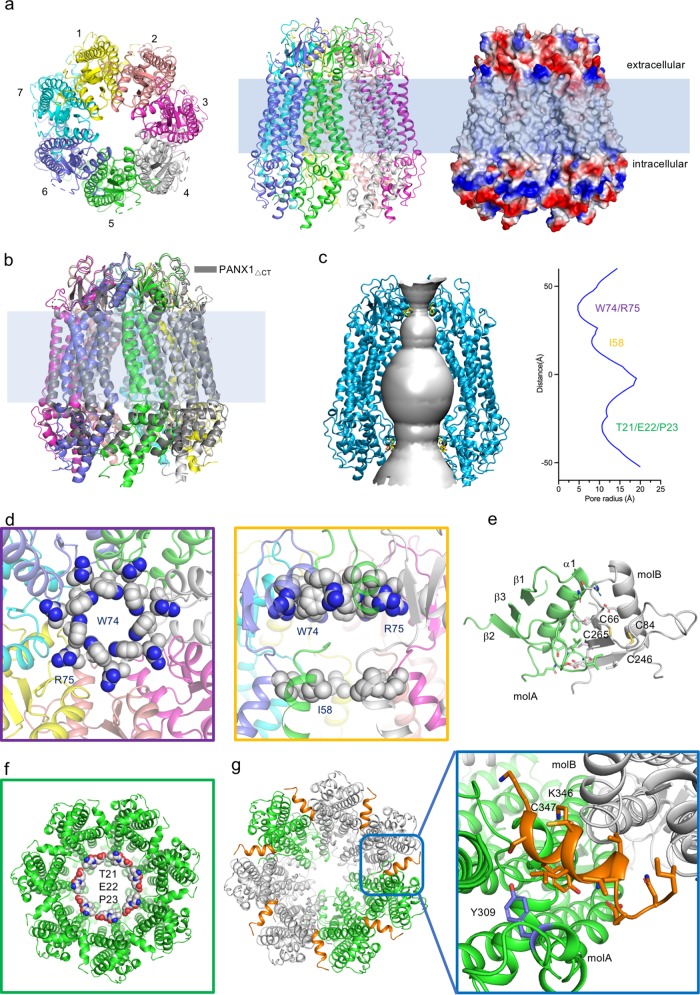
Structures of human PANX1. **a** Overall structure of human PANX1. Left, extracellular view; middle, side view; right, the surface electrostatic potential of PANX1 calculated by PyMOL. **b** Structural comparison between PANX1_ΔCT_ and full-length PANX1. Full-length PANX1 and PANX1_ΔCT_ are shown in cartoon and colored rainbow and gray, respectively. **c** The pore and pore radius of PANX1. Surface representation of the central pore of PANX1 (left panel); the pore radius of PANX1 was calculated by HOLE (right panel). **d** The narrowest constrictive site in the extracellular region. The key residues W74 and R75 are shown as spheres (left panel). Another constrictive site in the extracellular region, I58, is shown as spheres (right panel). **e** Interactions between two extracellular domains of PANX1. The residues in the interface of the two protomers are shown as sticks. **f** Intracellular constrictive site of PANX1. The key residues, T21/E22/P23, are shown as spheres. **g** Locations of gain-of-function mutations and posttranslational modification sites. The key helices are colored orange in the heptamer (left panel). K346 and C347 are shown as sticks. The phosphorylation site Y309 is colored purple.

Caspase 3-mediated cleavage of the cytoplasmic CT tail irreversibly activates PANX1 and triggers the release of the “find-me” signaling molecule for dying cell clearance.^1,6^ This cleavage-to-activation event was also observed in lipopolysaccharide-induced pyroptosis via caspase 11.^3^ Human PANX1 contains two identified caspase-cutting sites, site 1 (S1, DMRD) in the intracellular loop (IL) and site 2 (S2, DVVD) in the CT tail (Supplementary information, Fig. S1a). Cleavage at the S2 site is essential for the activation of PANX1.^1^ We generated CT-cleaved PANX1, named PANX1_ΔCT_, via *Drosophila* effector caspase (drICE) cleavage. As shown by SDS-PAGE, PANX1_ΔCT_ was partially cleaved by drICE at the S1 site, and this result is consistent with caspase 3 cleavage.^1^ However, the dramatically deferred monodisperse peak of PANX1_ΔCT_ observed by gel filtration (~1–1.5 mL) indicated that PANX1_ΔCT_ underwent a large conformational change or that reasonable disorder regions were removed (Supplementary information, Fig. S1b). To detect the activity of PANX1_ΔCT_, we performed protein reconstitution in a planar lipid bilayer and single channel electrophysiological assay in vitro (Supplementary information, Fig. S1c). Consistent with the patch clamp study,^7^ incorporation of wild-type PANX1 induced a small current opening of c.a. 3 pA, whereas incorporation of PANX1_ΔCT_ resulted in the channel opening up to 177 pA under +100 mV (Supplementary information, Fig. S1d). It is interesting that channel gating was observed in PANX1_ΔCT_ (Supplementary information, Figs. S1d and S7), with a closure of c.a. 29% of the fully opened channel.

This biochemical and electrophysiological investigation of PANX1 revealed that PANX1_ΔCT_ and full-length PANX1 adopt two distinct states, active and inactive states, respectively. We therefore collected the cryo-EM dataset for PANX1_ΔCT_ and solved its structure at 3.1 Å resolution following a similar image processing strategy as the full-length dataset (Fig. 1b; Supplementary information, Table S1 and Figs. S3 and S8). Surprisingly, the two structures revealed almost identical conformations except that the density of the intracellular region of PANX1_ΔCT_ was weaker (Fig. 1b; Supplementary information, Figs. S5 and S8). Thus, the structure of full-length PANX1 was employed for the following structural analysis due to its clearer density map.

The pore radius of PANX1 was calculated by HOLE.^10^ As the result shows, the radius of the narrowest constrictive site is ~4.7 Å, which is contributed by W74. The residue R75 also contributes to the positively charged ring (Fig. 1c, d). Interestingly, another constrictive site was uncovered at the extracellular site of the transmembrane domain. I58 from the tail of TM1 forms a larger hydrophobic ring (Fig. 1c, d). Importantly, the extracellular domain of each protomer provides an interface of the PANX1 oligomer (Fig. 1e). Each extracellular domain contains one helix, three β-sheets and two conserved disulfide bonds (Fig. 1e; Supplementary information, Fig. S6). The interface between the two domains is constituted by extensive hydrophobic and hydrogen-bond interactions, which further stabilized the constrictive sites (Fig. 1e).

The intracellular region of PANX1 comprises the N-terminus, the intracellular loop between TM2 and TM3, and the C-terminus (Supplementary information, Fig. S1a), which are essential for its regulation. We discovered a shrink ring formed by residues (T21/E22/P23) in the N-terminus (Fig. 1f; Supplementary information, Fig. S4). To validate whether the ring is involved in gating, three residues (21–23) of PANX1 were deleted, resulting in PANX1_Δ21–23_. The single channel conductance of PANX1_Δ21–23_ was recorded (Supplementary information, Fig. S9). As the result shows, incorporation of PANX1_Δ21–23_ into the bilayer induced constantly open channels without effective gating. However, the channel conductance of PANX1_Δ21–23_ was smaller than that of PANX1_ΔCT_ (Supplementary information, Fig. S9). Meanwhile, this result is consistent with gain-of-function mutations that cause aberrant PANX1 channel activity.^2^ Additional mutations of PANX1 also lead to channelopathy, including Δ392–426, K346E and C347S.^2^ Among them, Δ392–426 is similar to ΔCT, indicating that these residues are essential for the regulation of the CT tail. In heptameric PANX1, K346 and C347 are located in the helix between the two protomers (Fig. 1g; Supplementary information, Fig. S4). Moreover, the reported post-translational modification, phosphorylation at Y309 (Y308 in mouse), regulates the activity of PANX1.^11^ Interestingly, these residues are located in the same helix, named the inter-protomer helix (IH), or surrounding positions (Fig. 1g). The IH looks like a plug that is inserted between two protomers and blocks the potential movement of the intracellular regions (Fig. 1g). Therefore, the potential conformational changes of intracellular regions regulated by IH probably activate PANX1 channel.

The autoinhibition event facilitated by CT tails and the quantized mechanism for the activation of PANX1 have been proposed to account for the irreversible activation.^7,12^ However, disordered CT tails are missing in the structure of full-length PANX1. Does the CT tail interact with the residues in the pore? Given that the pore is more than 9 Å wide, an open conformation of PANX1 was probably captured. Interestingly, the channel gating was observed in PANX1_ΔCT_ (Supplementary information, Fig. S1d), which indicated PANX1_ΔCT_ was highly dynamic under the condition of electrophysiological study. However, it is still not sufficient to stabilize PANX1_ΔCT_ in a distinct conformation under the condition of cryo-EM sample preparation. In addition, PANX1 probably undergoes potential conformational changes regulated by IH. Therefore, more structural investigations and molecular dynamic simulation studies should be carried out to unveil other states of PANX1.

In general, our study sheds light on the gating mechanism of PANX1 channel. The identified extracellular and cytosolic shrink rings are essential for the activity of PANX1. Our structures also provide the foundation for the interpretation of related *PANX1* mutations. More importantly, PANX1 serves as a therapeutic target in opiate withdrawal^13^ and for drugs, such as spironolactone^14^ and probenecid.^15^ Our study could potentially promote structure-based therapeutic drug design.

The EM maps of the full-length and CT-cleaved PANX1 have been deposited in EMDB with accession codes EMD-0976 and EMD-0975, respectively. The corresponding atomic coordinates have been deposited in the Protein Data Bank with accession codes 6LTO and 6LTN.

## Supplementary information

Supplementary information
