# Supplementary material for: Structural basis for gating mechanism of Pannexin 1 channel
Source: Cell Res. 2020 Apr 13;30(5):452–4. doi: 10.1038/s41422-020-0313-x (PMC7196076; doi:10.1038/s41422-020-0313-x)
Supplement: Supplementary file 1 — Supplementary information [file 41422_2020_313_MOESM1_ESM.pdf]

Supplementary information

Materials and Methods

Cloning, expression and purification of human PANX1

Codon-optimized full length PANX1 was synthesized (Sangon Biotech) and cloned into modified pFastBac1 vector containing a C-terminal Strep-tag II (WSHPQFEK). Insect cell expression system was used to produce target protein, including virus generation and amplification. HighFive cell was infected by P2 virus at a cell density of 1.5-2.0×10^6 cells/mL and cultured at 27 °C for 72 h. Cells were collected by centrifugation at 1,500 g for 15 min, and resuspended in binding buffer consisting of 100 mM Tris pH 8.0, 150 mM NaCl and 1 mM EDTA. 2% (w/v) n-Dodecyl β-D-maltoside (DDM) was used to extract PANX1 with 0.8 μM aprotinin, 2 μM pepstatin A and 5 μg/mL leupeptin. After 1.5 h incubation at 4 °C, cell debris was isolated by centrifugation at 25,000 g for 45 min, and the supernatant was incubated with pre-equilibrated StrepTactin Sepharose High Performance resin (GE Healthcare) for 30 min. Then the suspension containing affinity resin and supernatant was loaded into gravity columns, and the resin was washed by 10 column volumes of binding buffer with 0.01% (w/v) lauryl maltose neopentyl glycol (LMNG, Anatrace) for 3 times. The full length PANX1 was eluted by 10 column volumes elution buffer (binding buffer with 0.01% LMNG and 2.5 mM desthiobiotin).

We generated CT-cleaved PANX1 (Δ380-426), named $PANX1_{\Delta CT}$, via *Drosophila*

effector caspase (drICE) cleavage. The drICE exhibits high homology and shares the same substrate specificity (DXXD) with mammalian caspases[1]. On-column digestion was carried out, which ensures that proteins were eluted from affinity column without the CT tail. For drICE-digested protein, drICE was added to resin suspension to a final concentration of 0.01 mg/mL and incubated at 4 °C for 2 h. Finally, drICE-digested PANX1 was eluted by 10 column volumes elution buffer without desthiobiotin. Eluted protein was concentrated and further purified by gel filtration (Superose 6 10/300 Increase column, GE Healthcare), buffered with 25 mM Tris pH 8.0, 150 mM NaCl and 0.01% LMNG. After identified by SDS-PAGE, peak fractions were collected for single channel recording or concentrated to approximately 8 mg/mL for cryo-EM sample preparation. All experiments were implemented at 4 °C.

Cryo-EM sample preparation and data acquisition

Aliquots of 3.5 μL concentrated protein were loaded onto glow-discharged holey carbon grids (Quantifoil Cu R1.2/1.3, 300 mesh). Grids were blotted for 3.5 s and plunge-frozen in liquid ethane cooled by liquid nitrogen using Vitrobot Mark IV (Thermo Fisher Scientific) at 8 °C with 100 percent humidity. Grids were transferred to a Titan Krios electron microscope operating at 300 kV and equipped with a Gatan Gif Quantum energy filter (slit width 20 eV). Micrographs were recorded using a K3 camera (Gatan) in super-resolution mode with a nominal magnification of 81,000x, resulting in a calibrated pixel size of 0.5435 Å. Each stack of 32 frames was exposed for 5.6 s, with an exposing time of 0.175 s per frame. The total dose for each stack was

about 50 $e^-/Å^2$. AutoEMation was used for fully automated data collection[2]. All 32 frames in each stack were motion corrected with MotionCor2[3], and dose weighting was performed [4], with 2-fold binned to a pixel size of 1.087 Å/pixel. The defocus values were set from -1.0 to -1.5 µm and were estimated by Gctf[5].

Image processing

The image processing procedures were shown in Fig S4 and Fig S8. A total of 2,541 and 1,828 micrograph stacks were collected, and 2,064,448 and 2,197,875 particles were auto-picked by Gautomatch (K. Zhang, www.mrc-lmb.cam.ac.uk/kzhang/) for full length and CT-cleaved dataset, respectively. All subsequent 2D and 3D classifications and auto-refinements were performed using RELION3.0[6]. After two rounds of reference-free 2D classification, 1,120,110 and 1,358,478 particles were selected for full length and CT-cleaved dataset, respectively. A subset of 100,000 particles was first applied to 3D classification with an initial model generated by RELION with C7 symmetry imposed. The best map from 3D classification was selected as the reference map for an auto-refinement of all selected particles. Then two rounds of local search 3D classifications were performed, each with several parallel runs in different class number (K=4 to 8). In the first round of local search 3D classification, an angular sampling of 1.8° was applied. The good particles from each run were selected and combined and duplicated particles were removed, yielding 397,740 and 381,458 particles for full length and CT-cleaved dataset, respectively. The selected particles were subjected to an auto-refinement, resulting in maps at 3.24 Å and

3.15 Å resolution, respectively. In the second round of local search 3D classification, an angular sampling of 0.9° was applied. A total of 251,945 and 133,330 selected particles from good classes were combined for auto-refinement, with local defocus values calculated with Gctf [5] for each particle. The final resolution of the 3D auto-refinement after post-processing with an overall soft mask was 3.06 Å for full length dataset and 3.10 Å for CT-cleaved dataset.

To further improve the map quality of the intracellular region, the final selected particles of the full-length dataset were applied symmetry expanding for another round of local 3D classification (Supplementary information, Fig. S4). A soft mask that covers a monomer was applied during the 3D classification with C1 symmetry. The best class with 1,298,807 particles were selected for 3D auto-refinement, yielding a reconstruction with a resolution of 3.34 Å. Although the reported resolution of this map was not as high as the overall map, it shows better map quality in the intracellular region and addresses extra density at the N-terminals. Therefore, modeling building of the intracellular region was mostly based on this map.

Reported resolutions are based on the gold-standard Fourier shell correlation (FSC) 0.143 criterion. Before visualization, all density maps were corrected for the modulation transfer function of the detector and sharpened by applying a negative B-factor that was estimated using automated procedures[7]. Local resolution variations were estimated using RELION.

Model building and refinement

The protomer of PANX1 was manually built in Coot[8]. Seven protomers were docked using PHENIX[9]. The model of full length PANX1 was docked in the density map of $PANX1_{\Delta CT}$ using PHENIX and followed by modification using Coot. The models were refined against the corresponding maps using PHENIX in real space (phenix.real_space_refine) with secondary structure and geometry restraints generated by ProSMART[10].

Overfitting of the overall models was monitored by refining the models in one of the two independent half maps from the gold-standard refinement approach and testing the refined model against the other map[11]. Statistics of 3D reconstruction and model refinement can be found in supplementary Table S1.

The EM maps of the full length and CT-cleaved PANX1 have been deposited in EMDB (www.ebi.ac.uk/pdbe/emdb/) with accession codes EMD-0976 and EMD-0975. The corresponding atomic coordinates have been deposited in the Protein Data Bank (www.rcsb.org) with accession codes 6LTO and 6LTN.

Bilayer reconstitution and single channel recording

A vertical lipid bilayer cup consisting of -cis and -trans chambers with an aperture of 150 μm between them was used for the recording (Warner Instruments). The -cis chamber was connected to grounding electrode, while the -trans chamber was connected to reference electrode. Buffer solution of 140 mM NaCl, 10 mM KCl, 10 mM HEPES, pH 7.5 or 150 mM KCl, 10 mM HEPES, pH 7.5 was used. A planar lipid bilayer was formed by painting *E. coli* polar lipid extract (Avanti, USA) in hexane

solution (25 mg/mL) through the aperture with air bubble from a pipette tip. 2 μL 0.5% LMNG detergent was added to the -cis side of the chamber to maintain protein solubility. Solution containing the wild-type or mutant PANX1 was added to the -cis chamber under 100 mV holding potential. The transmembrane current was recorded by Axon patch 700B amplifier (Molecular Devices) controlled by pCLAMP10 software (Molecular Devices). The signals were filtered to 10 kHz using an 8-pole low-pass Bessel filter (LPF-8, Warner Instruments) and digitized at a sampling rate of 20 kHz using a Digidata 1550B digitizer (Molecular Devices). The data was analyzed and plotted by Origin Pro 2019.

References

1 Fraser, A. G. & Evan, G. I. Identification of a Drosophila melanogaster ICE/CED-3-related protease, drICE. *EMBO J* **16**, 2805-2813, doi:10.1093/emboj/16.10.2805 (1997).

2 Lei, J. & Frank, J. Automated acquisition of cryo-electron micrographs for single particle reconstruction on an FEI Tecnai electron microscope. *Journal of structural biology* **150**, 69-80, doi:10.1016/j.jsb.2005.01.002 (2005).

3 Zheng, S. Q. *et al.* MotionCor2: anisotropic correction of beam-induced motion for improved cryo-electron microscopy. *Nature methods* **14**, 331-332, doi:10.1038/nmeth.4193 (2017).

4 Grant, T. & Grigorieff, N. Measuring the optimal exposure for single particle cryo-EM using a 2.6 A reconstruction of rotavirus VP6. *eLife* **4**, e06980, doi:10.7554/eLife.06980 (2015).

5 Zhang, K. Gctf: Real-time CTF determination and correction. *Journal of structural biology* **193**, 1-12, doi:10.1016/j.jsb.2015.11.003 (2016).

6 Zivanov, J. *et al.* New tools for automated high-resolution cryo-EM structure determination in RELION-3. *eLife* **7**, doi:10.7554/eLife.42166 (2018).

7 Rosenthal, P. B. & Henderson, R. Optimal determination of particle orientation, absolute hand, and contrast loss in single-particle electron cryomicroscopy. *J Mol Biol* **333**, 721-745, doi:10.1016/j.jmb.2003.07.013 (2003).

8 Emsley, P. & Cowtan, K. Coot: model-building tools for molecular graphics. *Acta crystallographica. Section D, Biological crystallography* **60**, 2126-2132, doi:10.1107/S0907444904019158 (2004).

9 Adams, P. D. *et al.* PHENIX: a comprehensive Python-based system for macromolecular structure solution. *Acta crystallographica. Section D, Biological crystallography* **66**, 213-221, doi:10.1107/S0907444909052925 (2010).

10 Nicholls, R. A., Fischer, M., McNicholas, S. & Murshudov, G. N. Conformation-independent structural comparison of macromolecules with ProSMART. *Acta crystallographica. Section D, Biological crystallography* **70**, 2487-2499, doi:10.1107/S1399004714016241 (2014).

11 Amunts, A. *et al.* Structure of the yeast mitochondrial large ribosomal subunit. *Science* **343**, 1485-1489, doi:10.1126/science.1249410 (2014).

Table S1 Data collection, refinement and validation statistics

	PANX1 (EMD-0976) (PDB 6LTO)	$PANX1_{\Delta CT}$ (EMD-0975) (PDB 6LTN)
**Data collection**		
EM equipment	Titan Krios	
Voltage (kV)	300	
Detector	Gatan K3	
Energy filter	Gatan GIF Quantum, 20 eV slit	
Pixel size (Å)	1.087	
Electron dose ($e^-/Å^2$)	50	
Defocus range (μm)	-1.0 ~ -1.5	
Data set	Full length	Cleaved
Number of images	2,541	1,828
**Reconstruction**		
Software	RELION3	
Number of used Particles	251,945	133,330
Symmetry	C7	
Final Resolution (Å)	3.1	3.1
Map sharpening B-factor ($Å^2$)	-120.0	-80.0
Accuracy of rotation (°)	1.584	1.537
Accuracy of translation (pixels)	0.714	0.689
**Model building and refinement**		
Model building software	Coot	Coot
Refinement software	Phenix	Phenix
**Model composition**		
Protein residues	2156	1708
Side chains	2121	1666
**Validation**		
R.m.s deviations		
Bonds length (Å)	0.007	0.006
Bonds Angle (°)	0.993	0.946
MolProbity score	1.47	1.45
Ramachandran plot statistics (%)		
Preferred	92.43	95.34
Allowed	7.57	4.66
Outlier	0	0

Supplementary information, Fig. S1

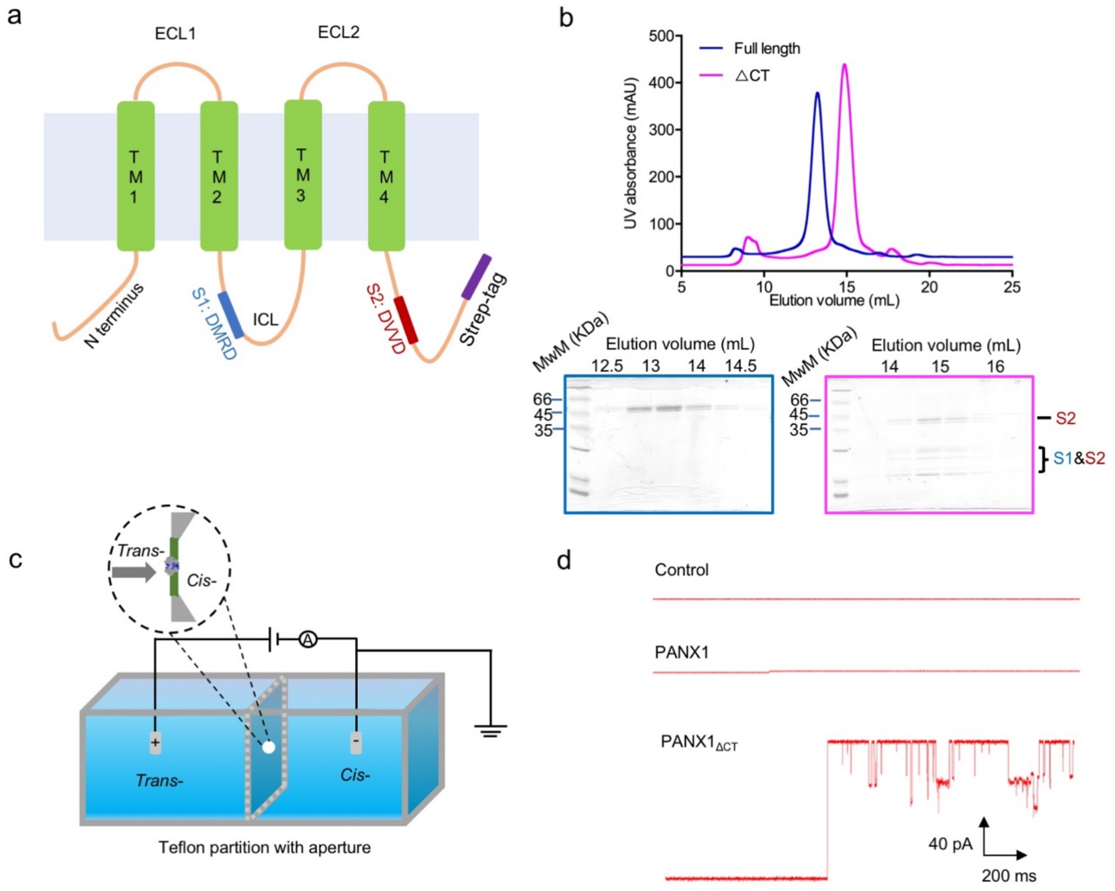

Fig. S1 purification and characterization of PANX1 and PANX1$_{\Delta CT}$. **a** Schematic of the full length PANX1. PANX1 contains two identified caspase-cutting sites (S1 in the ICL and S2 in the C terminus). **b** A representative size-exclusion chromatogram of purified full length PANX1 and PANX1$_{\Delta CT}$ (Superose 6 10/300 Increase). The peak fractions were applied to SDS-PAGE which was stained by Coomassie blue. In the SDS-PAGE, the proteins cleaved at only S2 and Both S1/S2 were labeled, respectively. **c** Diagram of single channel conductance recording setup. **d** Current recording traces of single channel conductance of PANX1 and PANX1$_{\Delta CT}$. Buffer condition: 140 mM NaCl, 10 mM KCl, 10 mM HEPES, pH 7.5. (n=3)

Supplementary information, Fig. S2

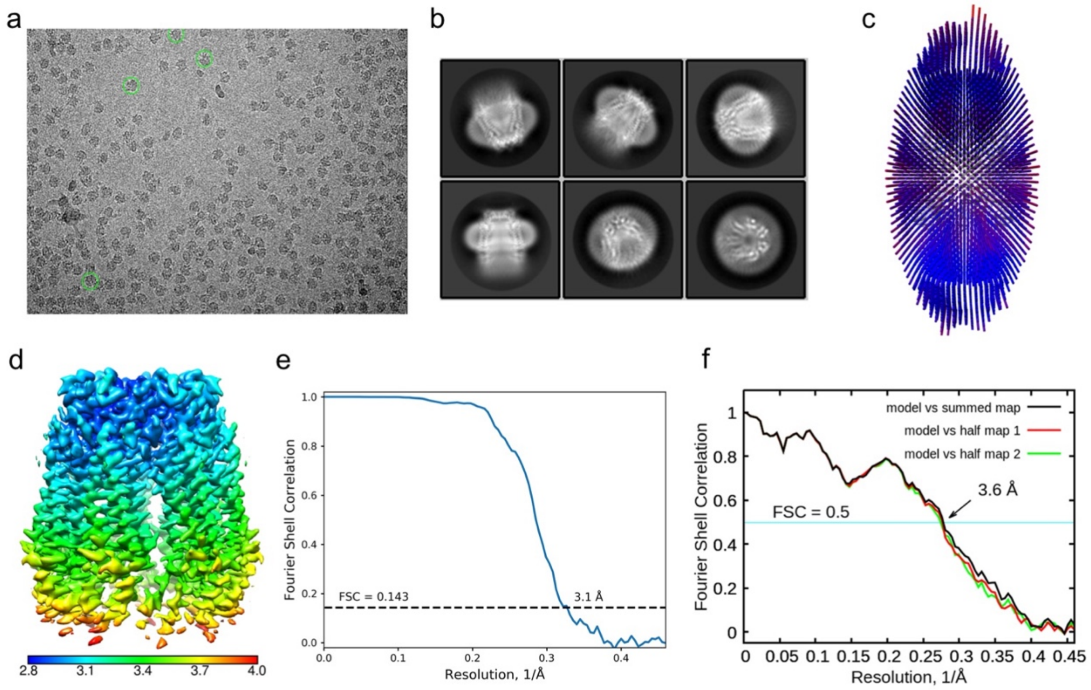

Fig. S2 The Cryo-EM analysis of full-length human Pannexin1. **a** A representative electron micrograph of full-length human Pannexin1. **b** Two-dimensional class averages of electron micrographs. Circle: 180 Å, box: 217.4 Å. **c** Angular distribution of 2D particles for the final reconstruction. The views are discretized and represent proportional to particles counts. **d** Overall EM density map of full-length human Pannexin1. **e** Gold-standard FSC curve for the density map generated using RELION. **f** Fourier shell correlation curves between reconstructed density and refined models.

Supplementary information, Fig. S3

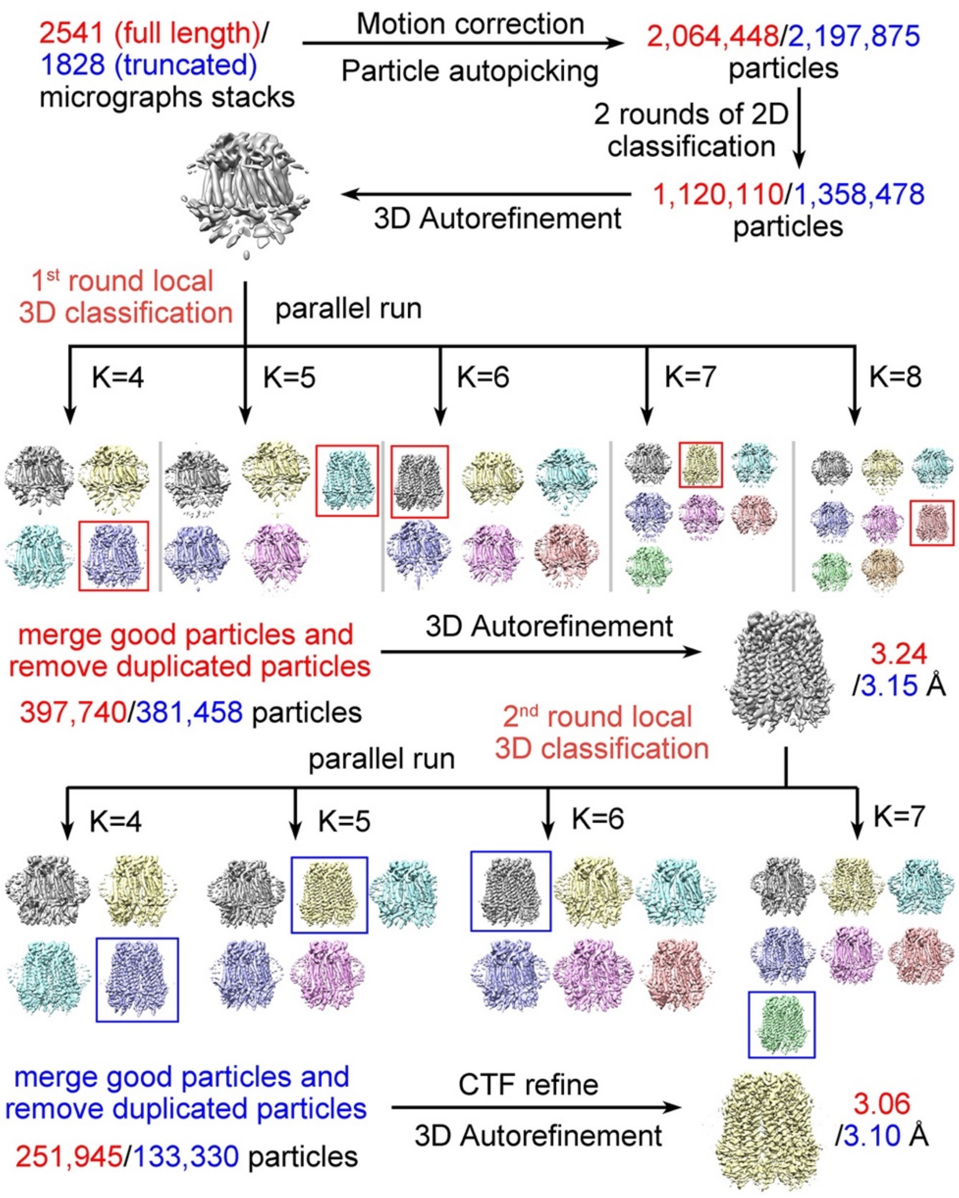

Fig. S3 Procedure of single particle data processing. Please refer to Materials and Methods section.

Supplementary information, Fig. S4

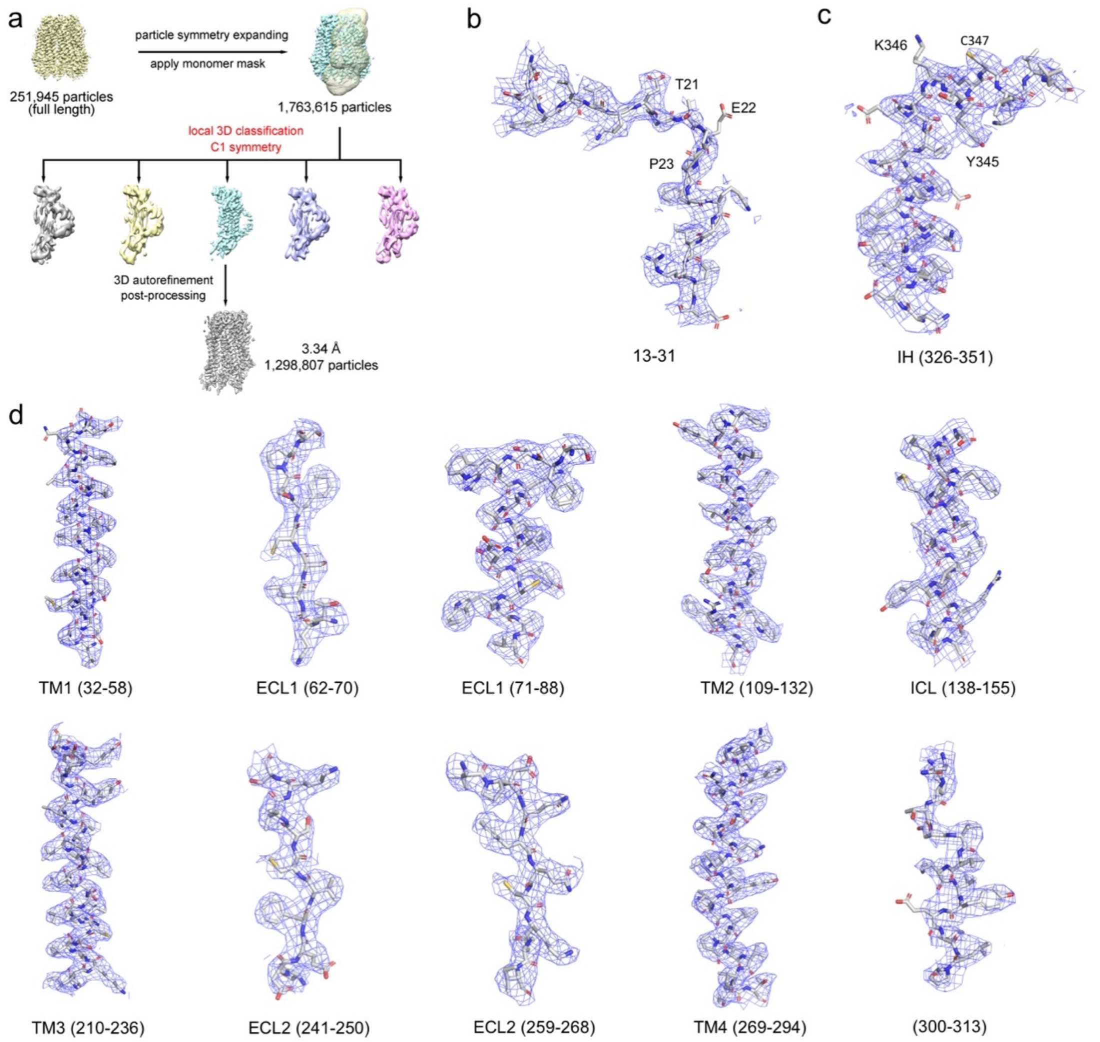

Fig. S4 Densities of important intracellular regions. **a** Procedure of density improvement of protomer. **b** Density of N-terminal loop region. **c** Density of IH. **d** Densities of ECLs, TMs and ICL. The densities were shown as blue mesh and the key residues were labeled.

Supplementary information, Fig. S5

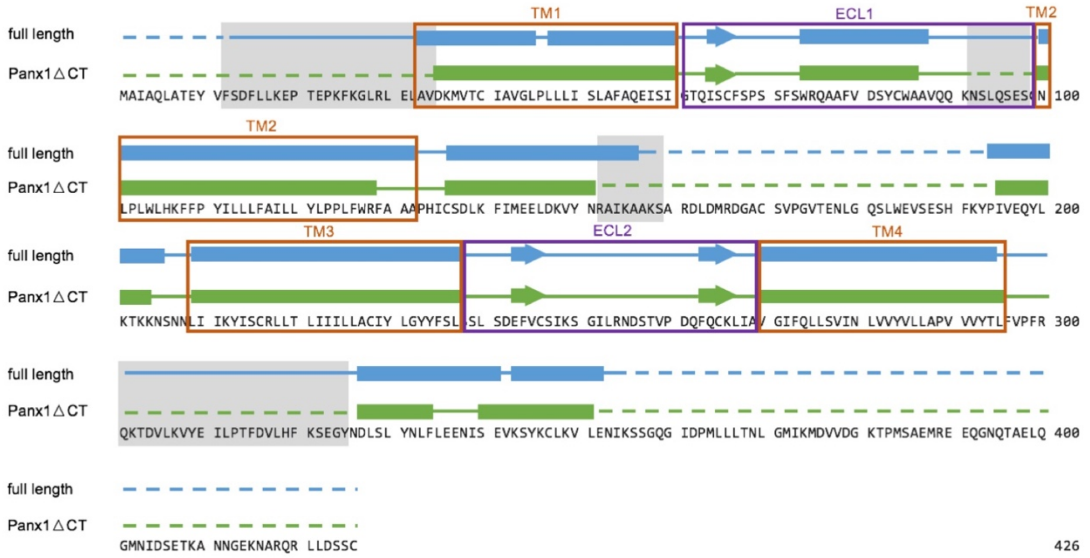

Fig. S5 Different sequences in models of two structures. The missing residues were labeled by dash line. Four different regions of two structures were highlighted by grey rectangles. Transmembrane helixes (TM) and extracellular loop (ECL) regions were highlighted by orange and purple, respectively.

Supplementary information, Fig. S6

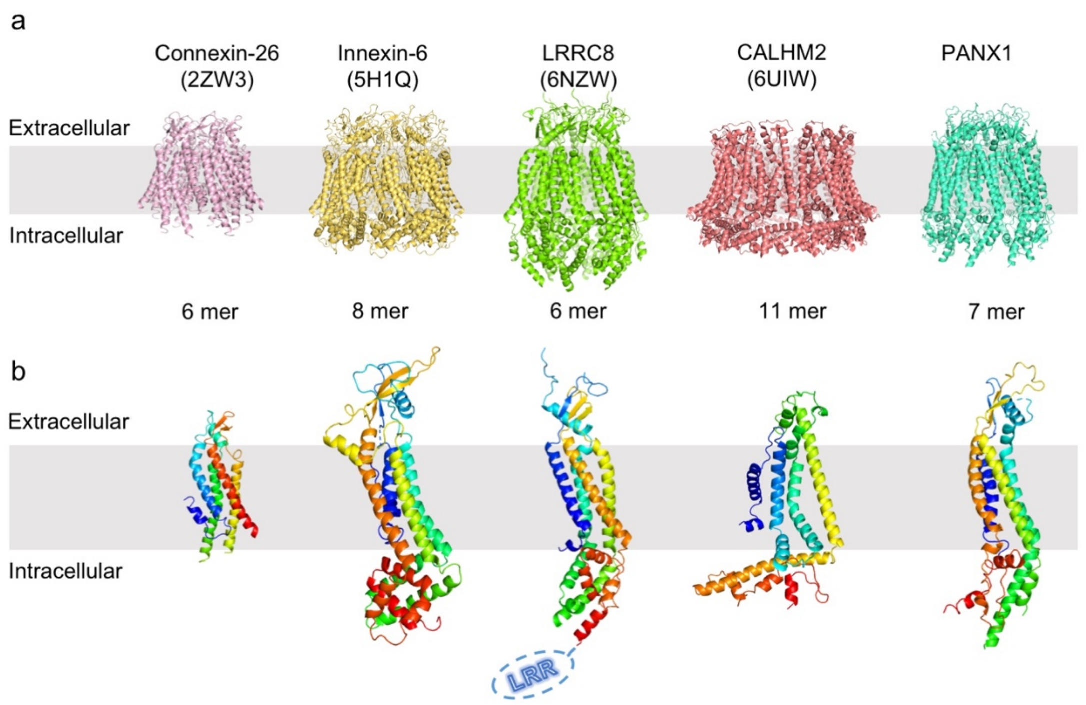

Fig. S6 Structures of gap junctions and other ATP-released channels. **a** Oligomeric structure of channels, including connexin26 (2ZW3), Innexin6 (5H1Q), LRRC8 (6NZW), CALHM2 (6UIW) and PANX1. **b** Structure of protomer of above channels.

Supplementary information, Fig. S7

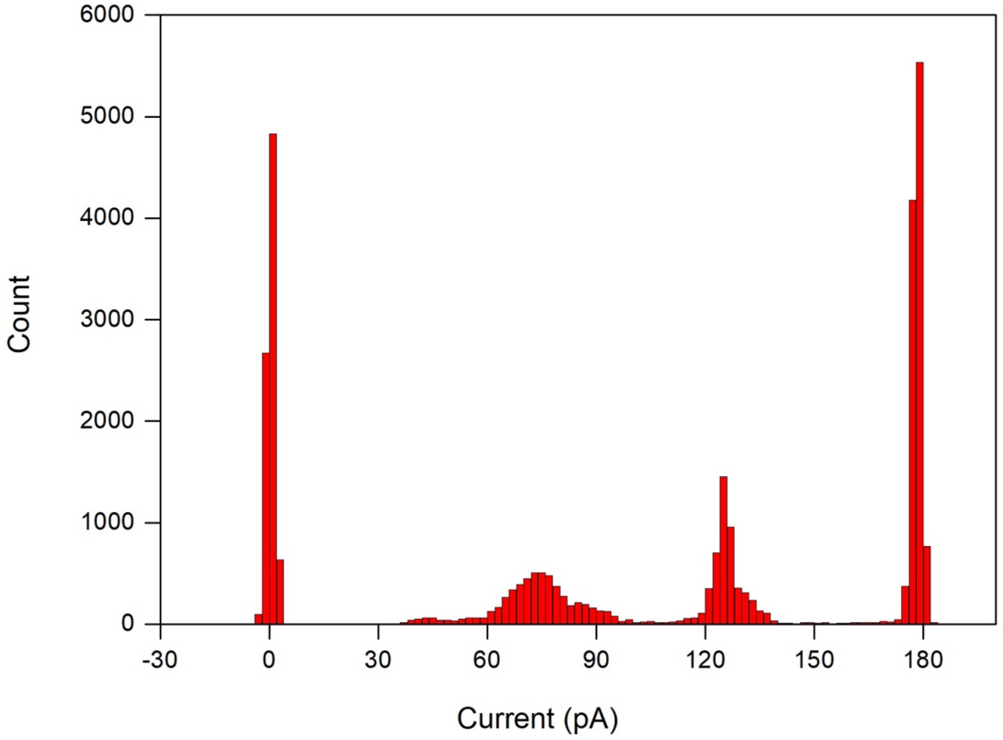

Fig. S7 Histogram of the gating from continuous current recording of a PANX1$_{\Delta CT}$ reconstituted in a planar lipid bilayer under +100 mV. Buffer condition: 140 mM NaCl, 10 mM KCl, 10 mM HEPES, pH 7.5.

Supplementary information, Fig. S8

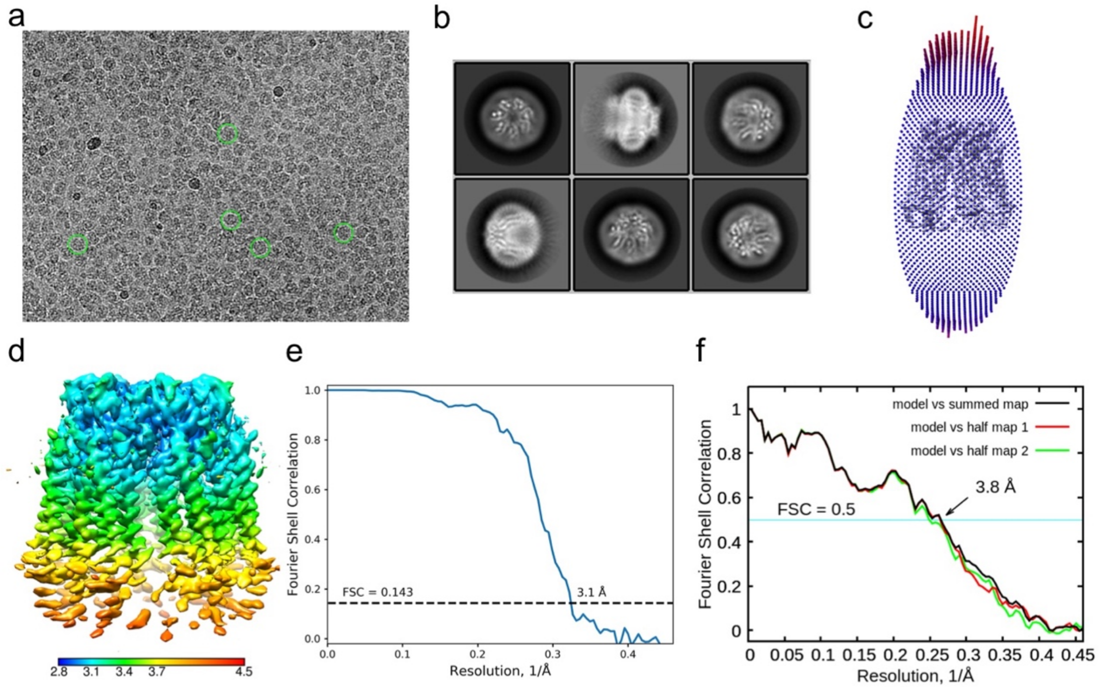

Fig. S8 The Cryo-EM analysis of CT-cleaved PANX1. **a** A representative electron micrograph of CT-cleaved PANX1. **b** Two-dimensional class averages of electron micrographs. Circle: 180 Å, box: 217.4 Å. **c** Angular distribution of 2D particles for the final reconstruction. The views are discretized and represent proportional to particles counts. **d** Overall EM density map of CT-cleaved PANX1. **e** Gold-standard FSC curve for the density map generated using RELION. **f** Fourier shell correlation curves between reconstructed density and refined models.

Supplementary information, Fig. S9

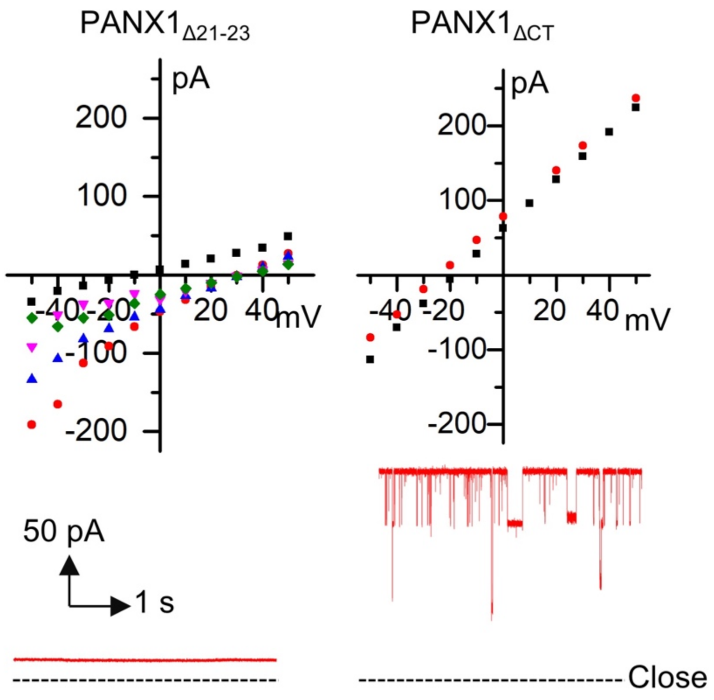

Fig. S9 Electrophysiological assay of PANX1$_{\Delta 21\text{-}23}$ and PANX1$_{\Delta CT.}$ Top: Current openings of PANX1$_{\Delta 21\text{-}23}$ and PANX1$_{\Delta CT}$ under a series of holding potentials. Bottom: Current traces under holding potential of +50 mV. Buffer condition: 150 mM KCl, 10 mM HEPES, pH 7.5.
